# Supplementary material for: In Silico Structural Prediction for the Generation of Novel Performant Midi-Dystrophins Based on Intein-Mediated Dual AAV Approach
Source: Int J Mol Sci. 2024 Sep 27;25(19):10444. doi: 10.3390/ijms251910444 (PMC11476470; doi:10.3390/ijms251910444)
Supplement: Supplementary file 1 [file ijms-25-10444-s001.zip › ijms-3218241-supplementary.pdf]

Figure S1

A

			Tot	Reduction
**H1**	Original hinges	QQVSIEAIQEVEMLPRPPKVTKEEHFQLHHQMHYSQQITVSLAQGYERTSSPKPRFKSYAYTQAAYVTTSDPTRSPFPSQHLEAPEDKSFGSSLME	96 aa	56,25%
	Shorten hinges	QQVSIEAIQEVEMLPRPPKVTKEEHFQLHHQMHKSFGSSLME	42 aa	
**H2**	Original hinges	AVTTTQPSLTQTTVMETVTTVTTREQILVKHAQEELPPPPPQKKRQITVD	50 aa	50%
	Shorten hinges	AVTTTQPSLTQTTVMETVTTVTTRE	25 aa	
**H3**	Original hinges	QPDLAPGLTTIGASPTQTVTLVTQPVVTKETAISKLEMPSSLMLEVP	47 aa	53,19%
	Shorten hinges	QPDLAPGLTTIGASPTQTVTLV	22 aa	
**H4**	Original hinges	AHRDFGPASQHFLSTSVQGPWERAISPNKVPYYINHETQTTCWDHPKMTELYQSLADLNNVRFSAYRTAMKL	72 aa	36,11%
	Shorten hinges	PNKVPYYINHETQTTCWDHPKMTELYQSLADLNNVRFSAYRTAMKL	46 aa	

B

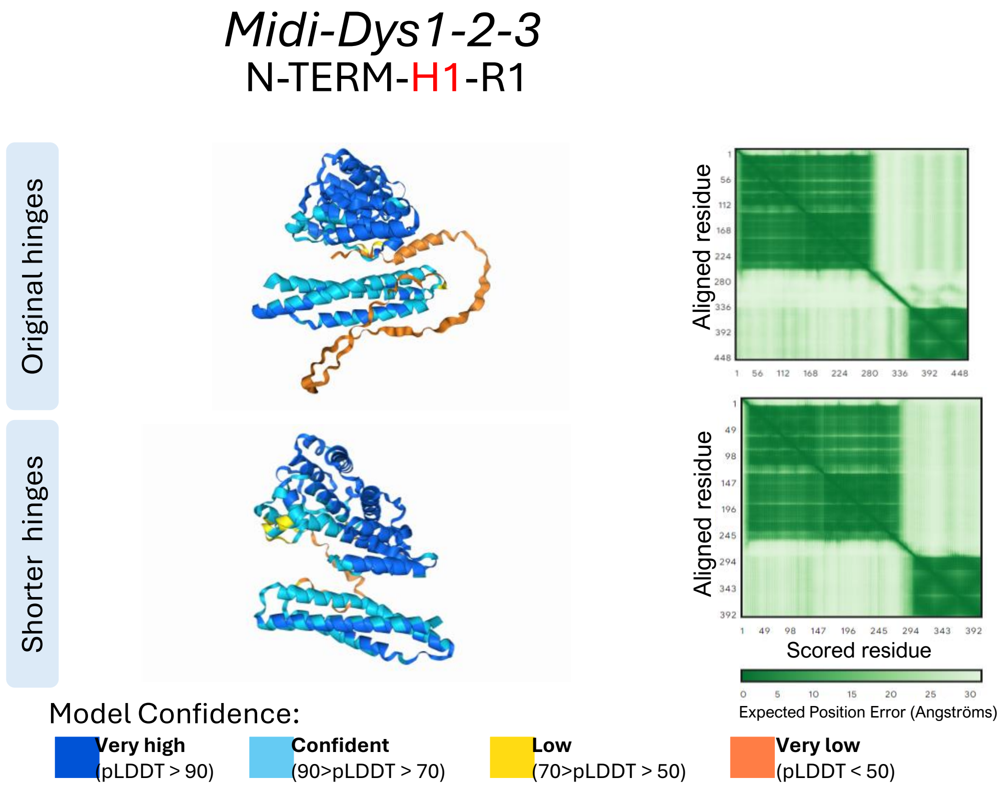

C

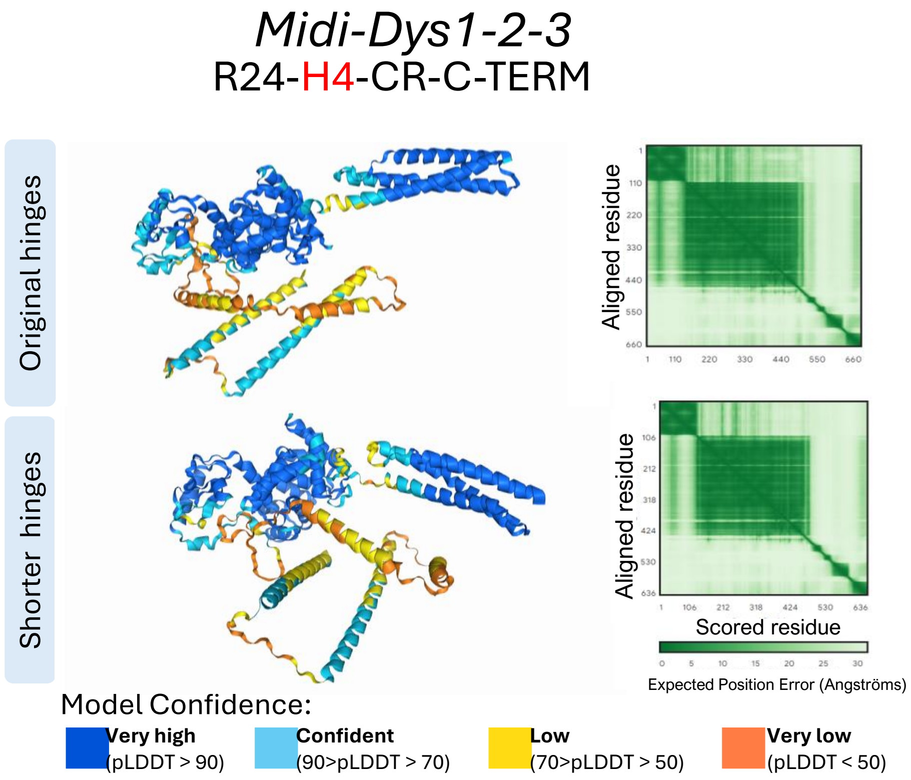

Figure S1. Design of Midi-dystrophins with shorten hinges. A) *Table representing the amino acids removed from the hinges (H). For each hinge, its original and shortened sequence is presented. The entity of the reduction is represented as a percentage compared to the original sequence.* ***B)*** *3D structure of the N-terminal region of Midi-Dys1/2/3, including the N-TERM (actin-binding domain 1), the hinge 1, and the spectrin-like region 1, with the original dystrophin hinge and after the shortening.* ***C)*** *3D structure of the C-terminal region of Midi-Dys1/2/3, including the R24, the hinge 4, the Cysteine-rich domain (CR) and the C-terminal (C-term) with the original dystrophin hinge and after the shortening.*

Figure S2

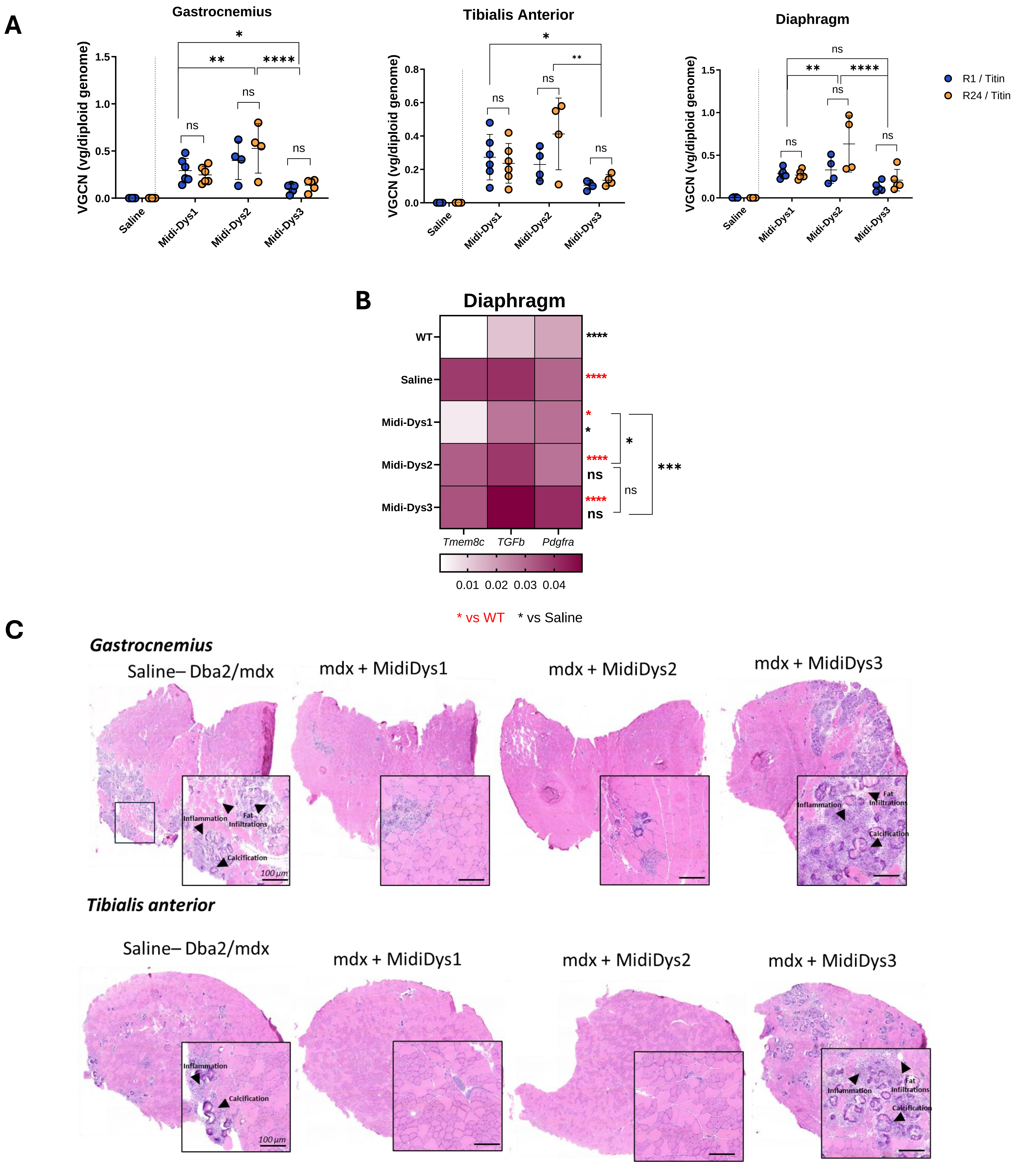

Figure S2. Viral copy number quantification of dual-vector Midi-Dystrophins and therapeutic efficacy in diaphragm of DBA2-mdx mice. A) *Viral genome copy number (VGCN) indicated as viral genome (vg) per diploid genome in gastrocnemius, tibialis anterior and diaphragm at 7 weeks post-injection in Dba2-mdx mice.* ***B)*** *Gene expression analysis of Tmem8c, TGF-β, and Pdgfr-α in diaphragm of WT mice and DBA2/mdx mice injected with saline, or Midi-Dys1, Midi-Dys2 and Midi-Dys3. Results are shown as heatmap of relative abundance of gene expression over the P0 normalizer gene.* ***C)*** *Hematoxylin Eosin staining in gastrocnemius and tibialis anterior of Dba2/mdx mice injected with Midi-Dys1, Midi-Dys2, Midi-Dys3 and Saline. Fat infiltration, inflammation and calcification areas are indicated with black arrows. For panel A: One-way ANOVA statistic test. *p<0.05, **p<0.005, ***p<0.001 ns= not significant. For panel B: Two-way ANOVA statistic test and multiple comparisons with main row effect. . *p<0.05, **p<0.005, ***p<0.001, ****<0.0001, ns= not significant.*
